# Consent for Genomic Epidemiology in Developing Countries: Added Human Subject Protection Also Needed

**DOI:** 10.1371/journal.pmed.0040214

**Published:** 2007-06-26

**Authors:** Robert Reinhard

The authors deserve thanks for laying out decent principles of communication [1]. But serviceable consent language is insufficient to address all issues of protection. That was the point of recent workshops held by the National Institutes of Health to develop a genome-wide association studies program [2].

Risks associated with personal identification may be incurred if information is subject to code breaking. Legal means are available to compel identification, including across national boundaries. Privacy protections under the Health Insurance Portability and Accountability Act (HIPAA) are subject to exceptions, including for law enforcement, downstream data users, or for other reasons, and are not available internationally. Even with authorization, the complexities associated with a repository may frustrate attempts to achieve meaningful comprehension. Use of data for purposes other than pharmaceutical product development or biomedical interventions would be an abuse resulting perhaps in travel restrictions or discrimination.

For these reasons, safeguards should be added, including:

- Amendments to prevent non-medical health access to personal identification information;
- Restrictions on recruitment of populations especially vulnerable to disclosure risks, such as prisoners or immigrants;
- Prohibitions on disclosure to or use by employers or third-party payors to deny medical coverage, assign differential premium risks, restrict access to therapies, or unfairly discriminate in employment.

Another risk from creation of a genomics repository is the potential for unjust stigmatization (see for example [3]). A workable program would state that the data are appropriate only for limited public health purposes involving product development or professionally derived biomedical intervention, and are insupportable for other use or by political or non-medical entities.

A researcher publishing results based on the genomic data should state affirmatively a boilerplate recognition of the abuse potential for stigmatization. This mechanism could prevent others from the wayward misappropriation of data for purposes other than those intended by professionals. The boilerplate could read:

“Conclusions derived from the genotypic or phenotypic characterization of individuals, groups, or families in this [publication] are meaningful or supportable only for the purpose of biomedical intervention or treatment and are unethical, insupportable, or inappropriate for use in other purposes. Use of the data to support any result of stigmatization, discrimination, or adverse social harm would constitute a misuse or abuse of the data.”

To increase the connection of benefits to participants, individuals should be given personal opportunities to receive news reports if they wish and learn of particular clinical trials directed at their characteristics. If the data are to be used in the development of pharmaceutical products, users should also be directed to plan and explain early on how targeted populations may have reasonable access to treatment or therapy if the product is successfully brought to market. These suggestions are consistent with the program outlined by Senator Barack Obama in the Genomics and Personalized Medicine Act of 2006 and Senator Olympia Snowe in the Genetic Information Nondiscrimination Act of 2007 [4,5].

Improved consent: Yes, but linked to and inseparable from strong protections and added benefits for participants.
